# Characterization of the mIF4G Domains in the RNA Surveillance Protein Upf2p

**DOI:** 10.3390/cimb46010017

**Published:** 2023-12-29

**Authors:** Edgardo M. Colón, Luis A. Haddock, Clarivel Lasalde, Qishan Lin, Juan S. Ramírez-Lugo, Carlos I. González

**Affiliations:** 1 Department of Biology, Río Piedras Campus, University of Puerto Rico, San Juan, PR 00931, USAclarivel.lasalde@upr.edu (C.L.); juan.ramirez3@upr.edu (J.S.R.-L.); 2 Molecular Sciences Research Center, University of Puerto Rico, San Juan, PR 00926, USA; 3 Department of Chemistry, University at Albany, Albany, NY 12222, USA; qlin@albany.edu; 4 RNA Epitranscriptomics and Proteomics Resource, University at Albany, Albany, NY 12222, USA

**Keywords:** *Saccharomyces cerevisiae*, codon, nonsense, nonsense-mediated mRNA decay, cloning, molecular, eukaryotic initiation factor-4G, aspartic acid

## Abstract

Thirty percent of all mutations causing human disease generate mRNAs with premature termination codons (PTCs). Recognition and degradation of these PTC-containing mRNAs is carried out by the mechanism known as nonsense-mediated mRNA decay (NMD). Upf2 is a scaffold protein known to be a central component of the NMD surveillance pathway. It harbors three middle domains of eukaryotic initiation factor 4G (mIF4G-1, mIF4G-2, mIF4G-3) in its N-terminal region that are potentially important in regulating the surveillance pathway. In this study, we defined regions within the mIF4G-1 and mIF4G-2 that are required for proper function of Upf2p in NMD and translation termination in *Saccharomyces cerevisiae*. In addition, we narrowed down the activity of these regions to an aspartic acid (D59) in mIF4G-1 that is important for NMD activity and translation termination accuracy. Taken together, these studies suggest that inherently charged residues within mIF4G-1 of Upf2p play a role in the regulation of the NMD surveillance mechanism in *S. cerevisiae.*

## 1. Introduction

Eukaryotic gene expression is highly regulated at multiple levels to ensure the proper synthesis of gene products. Among the myriad processes controlling gene expression there are several mechanisms, collectively known as mRNA surveillance, that ensure the quality of mRNA molecules. The most studied one is known as nonsense-mediated mRNA surveillance decay (NMD), which recognizes and targets mRNAs containing premature termination codons (PTCs) for degradation that would otherwise be harmful or toxic to the cell [1,2,3,4,5,6,7]. Along with the regulation of PTC-containing mRNAs, NMD has also been implicated in the regulation of some normal mRNA transcripts and long non-coding RNAs in yeast [8,9,10,11,12,13,14,15,16,17].

Upf1, Upf2, and Upf3 form the core complex of proteins necessary for NMD in eukaryotes. Yeast Upf2 is a 127 kDa acidic protein that accumulates in the perinuclear region of the cytoplasm [18,19,20]. It acts as an adapter protein when Upf1 binds to its C-terminal end and Upf3 to its N-terminal end [21,22,23,24]. Various models have been suggested to explain how these factors elicit NMD, which in turn results in the degradation of PTC-containing RNA [25,26,27,28,29,30,31,32,33,34]. One possibility that has been studied is that Upf1 ATPase and helicase activities are regulated by Upf1 interaction with Upf2. Before interacting with Upf2, Upf1 remains in a closed form that enhances it’s binding to RNA, which inversely minimizes its ATPase and helicase function [23,35,36,37]. When Upf2 binds, through its C-terminal, to the CH (rich in Cysteine and Histidine) domain of Upf1, it causes a conformational change in Upf1 to an open form, resulting in a reduction of its RNA binding activity while enhancing its ATPase and helicase function [35,38]. Much emphasis has been given to this N-terminal Upf1-Upf2 C-terminal interaction while the potential regulatory role of the Upf2 N-terminal has been neglected.

The Upf2 N-terminal region harbors three mIF4G domains like those found in the eukaryotic initiation factor eIF4G [39,40,41,42]. In yeast, the mIF4G-1 spans residues 1–310 from which residues 30–50 have shown to be critical for NMD [4,42]. Similarly, when mIF4G-1 or mIFG-2 are deleted in the human UPF2, individually or together, NMD activity is impaired [40]. The crystallized structure of yeast mIF4G-1 revealed that it is formed by 11 α-helices, where 5 pairs are ordered as antiparallel helices stacked against each other (h1–h10) with an additional α-helix (hA) located at the N-terminus of the domain [42]. Comparison of human Upf2 protein mIF4G-1 with yeast mIF4G-1 shows that they are similar, differing only in that the human mIF4G-1: (1) helix hA is much longer, (2) harbors an additional helix (h8i) inserted between helices h8 and h9, (3) has an extra α-helix (hB), and (4) the mIF4G-1 domain structure is more compact [40,42]. In yeast, this domain harbors phosphorylated residues S32 and S33 that have been shown to be required for NMD [4]. Basic residues K35, R36, K42, K43 and K45, and acidic residues D31 within the yeast mIF4G-1 domain have also been shown to be essential for NMD [4,42].

In this study, our central research question aimed to investigate the role of specific residues within the Upf2p mIF4G domains in regulating NMD and translation termination accuracy. Our hypothesis posited that these mIF4G domains are intricately involved in NMD regulation, suggesting that certain residues within these domains play a crucial role in NMD activity. To address this, Northern blot analysis and nonsense suppression assays were performed. Results showed a functional region of 10 amino acid residues withing each one of Upf2p mIF4G-1 and mIF4G-2 domains that are essential for both NMD and translation termination accuracy. We identified two novel phosphorylated residues in the 10-residue functional regions of mIF4G-1and mIF4G-2. Intriguingly, mutational analysis of these phosphorylated residues demonstrated that, contrary to expectations, they did not impair NMD activity.

Further characterization of these mIF4G domain functional regions showed that segments comprised of three or four residues at the positions L57–D59, Q421–W423 and K425–V428 are required for both NMD and translation termination accuracy. Specifically, a mutation of an aspartic acid (D59) within the mIF4G-1 domain impaired the activity of both mechanisms. This study sheds light on the specific residues crucial for the regulatory mechanisms of NMD and translation termination accuracy mediated by Upf2p mIF4G domains.

## 2. Materials and Methods

### 2.1. Strains and Plasmids

*S. cerevisiae* strain S288C derivative *upf2*Δ*(Mat ɑ*, *ura3-52*, *trp1-*Δ*1*, *leu2-2*, *tyr7-1*, *can1-100*, *his3-*Δ*200*, *upf2*Δ*1*: HIS3) was used for all the experiments performed in this study. The yeast 2 micron plasmid pG-1 containing the Flag-*UPF2* allele was used as the vector [43]. Deletions of *upf2* were constructed using the Flag-*UPF2* allele as a template, subjected to the single-step cloning method [44]. Briefly, a pair of 5’-phosphorylated inverse primers separated by the region to be deleted was utilized for amplification, ensuring that the two primers were similar in size. The PCR mixture comprised 150 ng of each primer (Integrated DNA Technologies, Coralville, IA, USA), 50 ng of template plasmid DNA, 200 mM dNTPs, 2.5U Pfu Turbo DNA polymerase (Stratagene, San Diego, CA, USA), and the buffer supplied with the polymerase in a total volume of 50 mL. PCR was executed under the following conditions: denaturation at 95 °C for 3 min, followed by 18 cycles of denaturation at 95 °C for 45 s, annealing at 62 °C for 1 min, and extension at 68 °C for 2 min/kb template.

Point mutants of *upf2* were constructed using Flag-*UPF2* allele as a template, subjected to the site-directed mutagenesis method [43]. Briefly, a single primer was employed to generate point mutations in a single linear amplification reaction. The PCR mutagenesis reaction was carried out in a reaction volume of 25 μL, comprising 1 μL of plasmid DNA, 200 nM of each primer, 200 μM dNTPs, and 1.25U Pfu enzyme in 1 × PfuDNA polymerase reaction buffer. The procedure commenced with a preliminary denaturation step at 95 °C for 3 min, followed by 18 cycles of PCR. These cycles involved 15 s of denaturation at 95 °C, 1 min of annealing at a temperature 2 °C higher than the melting temperature (Tm) of the primer head to enhance specificity of amplification, and 12 min of extension at 68 °C. All constructs were sequenced, and mutations were confirmed by sequence alignment. 

Yeast strains were transformed by an improved version of the lithium acetate method [45]. Briefly, cells inoculated into liquid YPD medium were allowed to grow overnight until reaching a concentration of 1–2 × 10^7^ cells/mL. The overnight culture was then diluted to 2 × 10^6^ cells/ mL in fresh, warm YPD, and regrown to a concentration of 1 × 10^7^ cells/mL. Subsequently, the cells were harvested, washed in sterile water, resuspended in 5.0 mL of sterile water, and transferred to 1.5 mL microfuge tubes where they were pelleted. Cells were then washed in 1.0 mL of TE/LiAc and resuspended at a concentration of 2 × 10^9^ cells/mL in 1 × TE/LiAc. In microfuge tubes, 50 μL of yeast cells were suspended, mixed with 1 μg of transforming DNA and 50 μg of single-stranded salmon sperm carrier DNA. A 300 μL sterile 40% PEG 4000 solution was added, and the mixture was incubated at 30 °C with agitation for 30 min. Following this, a heat shock was applied in a 42 °C water bath for 15 min. The mixture was then spun down in a microfuge for 5 s. The resulting cell pellet was resuspended in 1.0 mL of 1 × TE, appropriately diluted, and plated onto selective medium to identify successfully transformed cells.

### 2.2. Whole Cell Protein Extract and Western Blot

Cells were cultured in a synthetic liquid media lacking tryptophan to an optical density (OD_600_) of 1.0–1.2 followed by centrifugation at 3200 rpm for 10 min at 4 °C. Cells were then washed in: (1) 15 mL of cold ddH_2_O and centrifuged; (2) 15 mL of cold Buffer XA (20 mM HEPES, NaOH pH = 7.4, 150 mM NaCl, 2.0 mM EDTA, 0.01% Triton X-100, 30 mM Sodium Fluoride, 30 mM β-glycerol phosphate, 5 mM Sodium Pyrophosphate, 1 mM PMSF, 1X Protease Inhibitor Cocktail, 100 nM Okadaic Acid) and centrifuged; and (3) re-suspended in 5 mL of Buffer XA. Cells were then centrifuged, followed by the addition of 350–400 µL buffer XA and an equal volume of acid-washed glass beads. Vortex was then applied 13 times for 30 s with 45 s of cooling between steps. Centrifugation was applied for 30 min at 13,200 rpm and the lysate containing the protein extract was moved to a new tube. Bovine serum albumin (BSA) was used as a protein standard to quantify total protein extract. Proteins were resolved in 10% SDS-PAGE and transferred to a nitrocellulose membrane (Bio-Rad, Hercules, CA, USA). To detect Flag-*UPF2*, anti-Flag (Sigma-Aldrich, St. Louis, MO, USA) and anti-Mouse peroxidase conjugated (Sigma-Aldrich, St. Louis, MO, USA) were used as primary and as secondary antibody, respectively. Pab1p was used as a loading control and was detected using anti-Pab1p (Sigma-Aldrich, St. Louis, MO, USA). Exposure of the membranes was performed using SuperSignal West Dura chemiluminescent substrate (Thermo Fisher Scientific, Waltham, MA, USA) in a Molecular Imager^®^ ChemiDoc^ΤΜ^ XRS + with Image Lab^TM^ Software (v. 5.2) (BioRad, Hercules, CA, USA).

### 2.3. Upf2 Protein Purification

Upf2p was purified using anti-Flag M2 affinity gel (Sigma-Aldrich, St. Louis, MO, USA) as previously described [4]. Briefly, cell protein extract was performed as described above. Propylene chromatography columns were filled with 1 mL of anti-Flag M2 affinity gel (Sigma-Aldrich, St. Louis, MO, USA). 15 mL of 1X TBS-T were used twice to wash the beads and 1 mL-original volume of Buffer XB (20 mM HEPES, NaOH pH = 7.4, 150 mM NaCl, 2.0 mM EDTA, 0.01% Triton X-100, 30 mM Sodium Fluoride, 30 mM β-glycerol phosphate, 5 mM Sodium Pyrophosphate, 1 mM PMSF) was added to the anti-Flag beads. The beads were then added to the protein extracts in the column and incubated overnight at 4 °C with constant rotation. Afterwards, the beads were washed once with 3 mL of Buffer XB, three times with Buffer XB-2 (20 mM HEPES, NaOH pH = 7.4, 300 mM NaCl, 2.0 mM EDTA, 0.01% Triton X-100, 30 mM Sodium Fluoride, 30 mM β-glycerol phosphate, 5 mM Sodium Pyrophosphate, 1 mM PMSF), and four times with the Buffer XB. Beads were then incubated with 200 µL of Buffer XC (20 mM HEPES, NaOH pH = 7.4, 150 mM NaCl, 2.0 mM EDTA, 0.01% Triton X-100, 30 mM Sodium Fluoride, 30 mM β-glycerol phosphate, 5 mM Sodium Pyrophosphate, 1 mM PMSF, Flag Peptide 5 µg/µL) for 15 min at 4 °C. Elution 1 was collected followed by the addition of 300 µL of Buffer XC to the beads to collect elution 2. Finally, 300 µL of Buffer XC was added to the beads to collect elution 3. Elutions were resolved in 10% SDS-PAGE, stained with Coomassie Brilliant Blue (Sigma-Aldrich, St. Louis, MO, USA), and analyzed by Western blot. BSA was used as a protein standard to determine the protein concentration.

### 2.4. Tandem Mass Spectrometry Analysis

Prior to analysis, sample gel pieces from three different transformants expressing the Flag-*UPF2* allele were washed, reduced, alkylated, and in-gel tryptic digested. Additional analysis was performed by in-gel gluc-c digestion. Proteolytic peptides were extracted from the gel. The phosphopeptides were enriched by TiO_2_ TopTip (Glygen Corp., Columbia, MD, USA). Both flow-through and elute were analyzed by LC-MS/MS, respectively. For this analysis, an ABSCIEX QSTAR XL mass spectrometer (AB Sciex, Framingham, MA, USA) was used. This MS was coupled to a CapLC (Waters Corp., Milford, MA, USA) HPLC with a Phenomenex C18 (3 μm, 300 A, 100 μm ID × 150 mm, Phenomenex, Torrence, CA, USA) analytical column, and a Vydac Everest C18, 300 A, 5 μm trap column. The solvents used were composed of 5% CH_3_CN + 0.1% formic acid + 0.01% TFA (Solvent A) and 85% CH_3_CN + 10% isopropanol + 5% H_2_O + 0.1% formic acid + 0.01% TFA (Solvent B). Samples were kept at a 250nl/min flow rate and a 60 min linear gradient from 10% to 100% B.

### 2.5. Mass Spectrometry Data Analysis

MS/MS spectra files were processed and combined using Mascot distiller software from MatrixScience with a processing macro that smooths, centers, and assesses the quality of data. In-house MASCOT 2.3 from Matrix Science (London, UK) was used to assist the interpretation of tandem mass spectra against targeted protein sequences. Error Tolerant Search was used in the analysis. Spectra which contained adequate information that remained unmatched were subjected to an Error Tolerant Search. All database entries from a first pass search that contained one or more peptide matches with scores at or above the homology threshold were selected for an error tolerant, second pass search. In this second pass search the complete list of modifications were tested. The list of modifications used by Mascot was taken directly from the Unimod database. The entries on the modification list were tested serially, and all permutations of each individual modification were analyzed. Mass delta of the modification was rejected if they were less than the smaller of the precursor mass tolerance and the fragment mass tolerance.

### 2.6. RNA Isolation and Northern Blot

Total RNA was isolated using the hot phenol method [46]. Briefly, cell cultures were grown to an OD_600_ of 0.8–2.0 in synthetic minimal medium and harvested by centrifugation at 4000× *g* for 10 min. The cells were suspended in 10 mL of ice-cold 50 mM sodium acetate (pH 5.3), 10 mM EDTA, after which 1 mL of 10% SDS was added. The cell suspension was vortexed and then extracted with an equal volume of phenol (previously equilibrated with 50 mM sodium acetate, pH 5.3, 10 mM EDTA) at 65 °C for 4 min. The mixture was rapidly chilled in a dry ice-ethanol bath until phenol crystals appeared. Following centrifugation at 4000× *g* the phenol phase was removed, leaving the interface intact. A second aliquot of phenol was added and the extraction was repeated. The aqueous phase was transferred to a new tube and extracted once with a 1:1 mixture-of phenolchloroform at room temperature for 5 min. The aqueous phase was then brought to 0.3 M sodium acetate, pH 5.3, and 2.5 vol of ethanol was added to precipitate RNA. RNA samples were suspended in sterile water and RNA concentration was determined by A_260_ after incubation at 65 °C for 5 min. Random-primed DNA probes were prepared from a 0.6-kb EcoRI-HindIII fragment spanning a region of the *CYH2* mRNA. Northern blots were quantified using a BioRad Molecular Imager FX. The activity of NMD was calculated by comparing the ratio of pre*-CYH2* to *CYH2* in *upf2*∆ strains transformed with either a vector (0%) or wild-type *UPF2* (100%). Values of mRNA level represent averages ± standard deviation from three independent experiments.

### 2.7. can1-100 Nonsense Suppression Assay

A canavanine drug sensitivity test was performed as previously described [47]. Briefly, wild-type and mutant strains were grown to mid-log phase (OD_600_ = 0.8–0.9) in synthetic liquid media lacking arginine and tryptophan. Samples from these cultures were serially diluted (1:10) five times and aliquots of the four dilutions were spotted on synthetic plates lacking both tryptophan and arginine without or with 200 μg and 250 μg of canavanine. Plates were incubated at 30 °C for 48 h.

### 2.8. can1-100 Nonsense Suppression Assay Growth Curves

Growth curves were performed using a Synergy H1 Hybrid Multi-Mode Microplate Reader (BioTek Instruments, Winooski, VT, USA). Briefly, wild-type and mutant strains were grown to mid-log phase at 30 °C in synthetic liquid medium lacking arginine and tryptophan and diluted to an OD_600_ = 1.6. From there, cells were plated into a Falcon 96 well clear flat bottom untreated cell culture microplate (Corning Inc., Corning, NY, USA) at a final OD_600_ = 0.25, and 500 μg of canavanine were added. Measurements were taken every hour, with an initial 0 h reading, for 36 hours under continuous high orbital shaking. The shaking speed was set to fast and frequency set to 559 (1 mm amplitude). The temperature was set at 30 °C. Cell growth was assessed by absorbance measurements made at 600 nm. Reads were collected using Gen5™ Data Analysis Software (v.2.0) (BioTek Instruments, Winooski, VT, USA). Growth curves were generated using GraphPad.

## 3. Results

### 3.1. Identification of Novel Phosphorylation Sites in Yeast Upf2p mIF4G Domains

**Figure 1:**
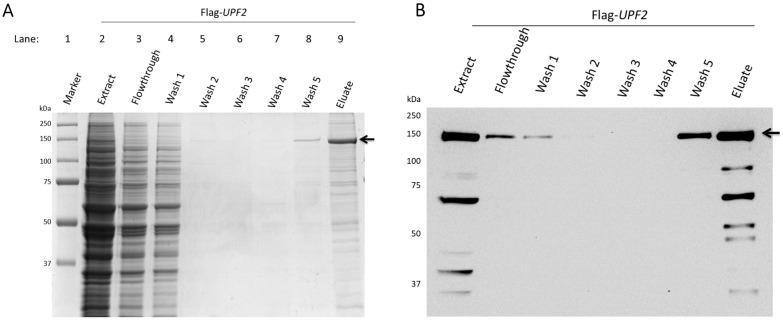
Upf2p was purified by affinity chromatography. (**A**) Coomassie blue-stained 10% SDS-PAGE of immunopurified Flag-Upf2p which is depicted by an arrow (←). (**B**) Western blot of immunopurified Flag-Upf2 protein depicted by an arrow (←).

**Figure 2:**
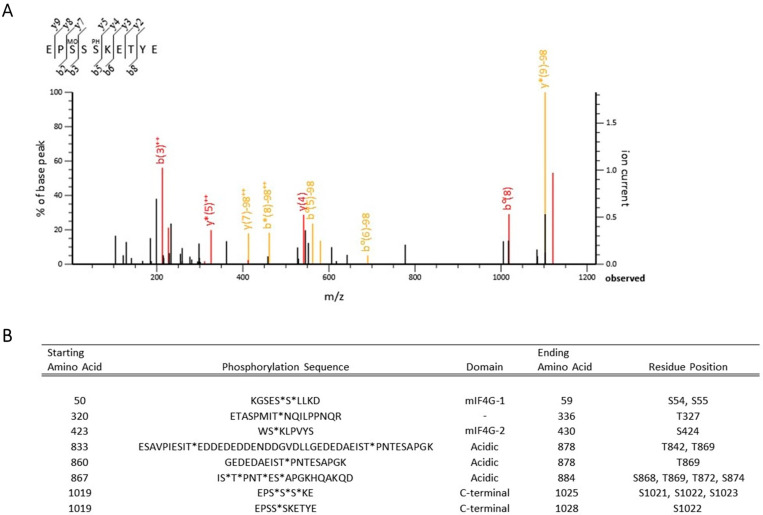
Novel phosphorylation sites were identified in Upf2p mIF4G-1 and mIF4G-2 domains. (**A**) Example of MS/MS spectrum for precursor ion EPSSSpKETYE. Precursor ion was selected and subjected to fragmentation, generating *b* and *y* product ions that represent specific fragments used for identification of the peptide sequence and phosphorylation sites. Loss of H_3_PO_4_ from *y5* fragment after β-elimination was evidently observed. The double plus sign (++) denotes the charge of the product ion, whereas the asterisk (*) denotes a modification or variation from the usual fragment. (**B**) Identified phosphorylation sites from the Upf2p precursor ions analyzed by mass spectrometry. First and fourth columns state the starting and ending residues of the precursor ions, respectively. Phosphorylated residues in the second column are depicted by an asterisk (*). Domains containing the peptides are specified in the third column. Last column shows the phosphorylated residue position within Upf2p.

Previous studies demonstrated that Upf2p is phosphorylated in *S. cerevisiae* and several phosphorylated residues were identified at the mIF4G-1 domain [4]. To identify additional novel phosphorylation sites in Upf2p mIF4G domains, the protein was purified from a *upf2*∆ *S. cerevisiae* strain expressing a Flag-tagged *UPF2* allele within a plasmid. Purification was performed in the presence of phosphatase inhibitors to avoid the loss of phosphorylation from residues. The purified samples were resolved by 10% SDS-PAGE and stained with Coomassie blue (Figure 1A). Flag-Upf2p migrated at approximately 127 kDa (Figure 1A, lane 9), consistent with previous reports [4,48]. Western blot analysis confirmed that the prominent band observed in the SDS-PAGE was indeed Flag-Upf2 (Figure 1B). The Coomassie-stained band harbouring Upf2p (~127 kDa) was excised and subjected separately to trypsin and gluc-c digestion. The proteolytic peptides obtained were eluted and subjected to mass spectrometry analysis (Figure 2A and Figure S1), as outlined in the materials and methods section. This analysis identified eight phosphorylated peptides, uncovering a total of twelve novel phosphorylated residues in Upf2p (Figure 2B).

**Figure 3:**
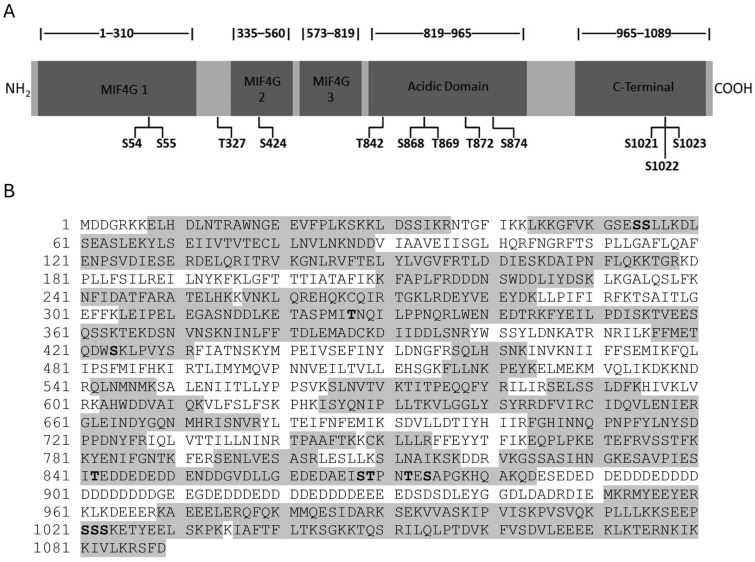
Three phosphorylation sites were identified in Upf2p mIF4G domains. (**A**) Illustrative representation of Upf2p showing the locations of the MIF4G-1 domain, MIF4G-2 domain, MIF4G-3 domain, acidic domain, and C-Terminal domain. The 12 phosphorylated residues identified by mass spectrometry are presented. (**B**) *S. cerevisiae* Upf2p amino acid sequence. Sixty-four percent (64%) of sequence coverage was obtained with the mass spectrometry analysis. Analyzed sequences are shown in gray and phosphorylated residues are in bold.

Previously suggested phosphorylated residues S32 and S33 [4] were not identified as phosphorylated, although various peptide fragments harboring these residues were analyzed. This may have been due to loss of phosphorylation during protein extraction, protein purification, or mass spectrometry analysis of Upf2p. Overall, twelve novel residues were identified as phosphorylated in *S. cerevisiae* Upf2p, of which three are in mIF4G domains: two in the mIF4G-1, and one in the mIF4G-2 (Figure 2B and Figure 3A). A total of 698 of the 1089 amino acids in *S. cerevisiae* Upf2p were present among the analyzed peptides (64% sequence coverage) (Figure 3B).

**Figure 4:**
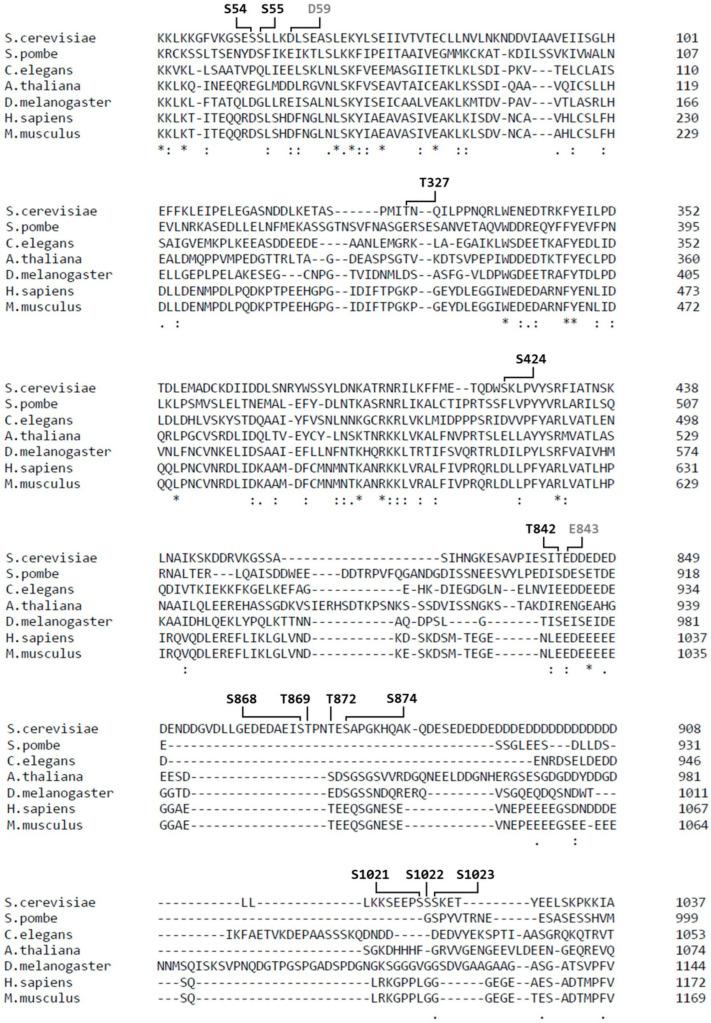
Several residues within Upf2 show a strong conservation of their negative charge. Alignment of sequences from Upf2 *S. cerevisiae* (NCBI NP_011944.2), *S. pombe* (NCBI NP_593784.1), *A. thaliana* (NCBI NP_181459.4), *C. elegans* (NCBI NP_500974.2), *D. melanogaster* (NCBI NP_572434.1), *H. sapiens* (NCBI NP_056357.1), and *M. musculus* (NCBI NP_001074601.1) shows a conservation of the negative charge of residues D59 and E843 as depicted by the colon (:). Phosphorylated residues are depicted in black while nearby negative residues are in gray. Alignment was constructed with Clustal O (version 1.2.4).

Sequence alignment showed that none of the Upf2p phosphorylated residues are conserved between species (Figure 4). However, phosphorylation of residues S54, S424 and T842 in *S. cerevisiae* showed a tendency to maintain a negative charge at those positions when compared to human Upf2 (Figure 4). Residue S54 is located within the mIF4G-1, S424 is located within the mIF4G-2 domain (Figure 3A), while residue T842 is in the acidic domain (Figure 3A). More importantly, the amino acid D59 (adjacent to the phosphorylated residue in the N-Terminal mIF4G-1 domain) and E843 (adjacent to the phosphorylated residue in the acidic domain) showed a conservation among species of strongly similar properties, namely their acidity given by the negative charge, suggesting an important role for these residues in the biological activity (Figure 4). Interestingly, both the mIF4G-1 and acidic domains have been shown to be necessary for NMD activity [4,42].

### 3.2. Upf2p mIF4G Domain Regions Harboring Phosphorylated Residues Are Required for NMD and Translation Termination Accuracy

**Figure 5:**
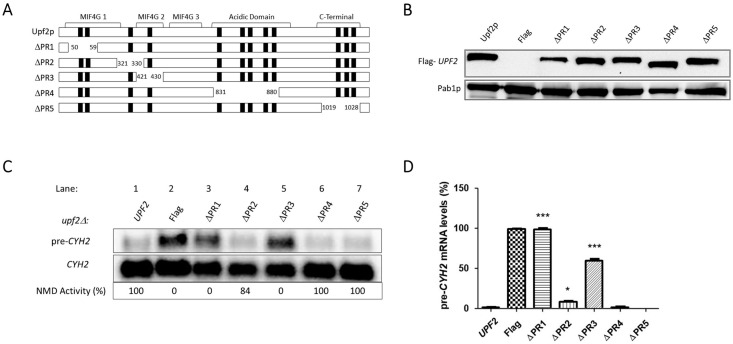
NMD activity requires Upf2p mIF4G-1 and mIF4G-2 phosphorylated regions 1 and 3. (**A**) Illustrative representation of Upf2p phospho-region mutants (ΔPR). Phosphorylated residues are depicted as black rectangles. In the phospho-region 1 (ΔPR1) residues K50–D59 were deleted, in phospho-region 2 (ΔPR2) residues E321-I330 were deleted, phospho-region 3 (ΔPR3) residues Q421-S430, in phospho-region 4 (ΔPR4) residues G831–Q880 were removed, and in phospho-region 5 (ΔPR5) residues E1019-E1028 were removed. (**B**) Analysis of cytoplasmic extracts by Western blot showing Upf2p expression. Poly(A) binding protein (Pab1p) was used as a loading control. (**C**) Northern blot from total cellular RNA was used to determine NMD activity of the Upf2p phospho-region deletions. (**D**) *pre-CYH2* mRNA accumulation from Northern blot expressed as a mean value ± standard deviation. Significance of results, when compared to the WT strain, are represented by asterisks (* *p* < 0.05, *** *p* < 0.001).

To assess the role of Upf2p phosphorylation, the phosphorylated residues were grouped and centered within five different regions. Except for one, each region consisted of ten residues and were separately deleted from the Flag-*UPF2* allele plasmid vector (Figure 5A). The phospho-regions were: 1 (K50–D59), 2 (E321–I330), 3 (Q421–S430), 4 (G831–Q880), and 5 (E1019–E1028). Only phospho-region 1 is located in the mIF4G-1; phosphor-region 2 is located in the region between mIF4G-1 and mIF4G-2 and phospho-region 3 is located within the mIF4G-2 domain [4,5] (Figure 5A). 

Western blot analysis confirmed the expression of these *upf2* mutants (Figure 5B). Northern blot analysis was used to determine NMD activity of these mutant strains by detecting the premature termination codon containing transcripts *CYH2* pre-mRNA, which encodes the ribosomal protein L29 [49] and *can1-100* mRNA which encodes an arginine amino acid membrane transporter [50]. *CYH2* pre-mRNA contains the in-frame premature termination codon [51] which, in conjunction with the mature *CYH2* mRNA, provides a ratio that has been routinely used as a measurement of NMD activity [4,51,52]. Results from total cellular RNA analysis from WT and phospho-region 2 (E321–I330), 4 (G831–Q880), and 5 (E1019–E1028) mutant strains showed that NMD activity was not affected in comparison to the defect observed in the *upf2*Δ strain (Figure 5C,D). This demonstrated that none of these deletions affected NMD activity. But deletion of phospho-region 1 (K50–D59) and phospho-region 3 (Q421–S430) showed reduced NMD activity like that of the *upf2*Δ strain, demonstrating that these deletions affected NMD activity (Figure 5C,D). Phospho-region 1 of Upf2p harbors the phosphorylated residues S54 and S55, while phospho-region 3 of Upf2p harbors the phosphorylated residue S424 (Figure 4).

**Figure 6:**
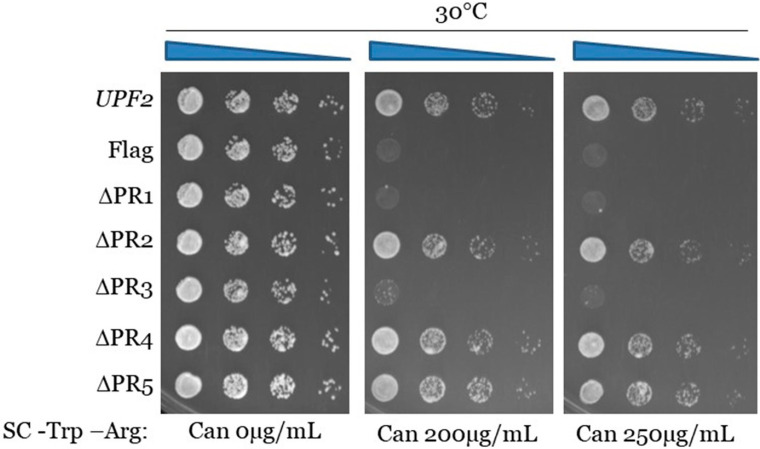
Proper translation termination requires Upf2p mIF4G-1 and mIF4G-2 phosphorylated regions 1 and 3. *can1-100* nonsense suppression assay was used to assess the role of Upf2p in translation termination efficiency. Wild-type and mutant *upf2* yeast strains were serially diluted (1:10) five times. Each yeast strain was spotted on SC-Trp-Arg plates, each supplemented with varying concentrations of canavanine (0, 200, or 250 µg/mL), followed by incubation at 30 °C for a duration of two days.

In addition to being critical for NMD, Upf2p also promotes translation termination [47]. The *can1-100* nonsense suppression assay was used to assess the role of Upf2p phospho-region 1 and phospho-region 3, both qualitatively and quantitatively, in translation termination efficiency. This assay exploits the fact that in a *can1-100* strain defective in translation termination, read-through of the nonsense codon produces a functional form of the protein Can1p, an arginine permease capable of importing the toxic arginine analog canavanine. Consequently, lower growth rates of the different Upf2p mutant strains in comparison to the WT are used to measure enhanced nonsense suppression activity [47,53,54]. Results from the *can1-100* nonsense suppression assay demonstrated that, like the wild-type *UPF2* strain, strains with deletions of phospho-region 2, phospho-region 4, and phospho-region 5 grow normally and thus do not show a nonsense suppression phenotype (Figure 6). In contrast, strains harboring the deletion of phospho-region 1 and phospho-region 3 show less growth in the presence of canavanine, suggesting a reduction in translation termination efficiency (Figure 6). In addition, strains with deleted phospho-region 1 and phospho-region 3 grown in synthetic complete liquid media with canavanine over a period show decreased growth, also indicating that these phospho-regions play a role in translation termination efficiency (Figure S2). Taken together, these data suggest that two small regions of Upf2p consisting of ten residues and harboring phosphorylated residues are critical for NMD activity as well as a proper translation termination.

### 3.3. Upf2p mIF4G-1 S54 and S55, and mIF4G-2 S424, Phosphorylated Residues Are Not Required for NMD and Translation Termination Accuracy

**Figure 7:**
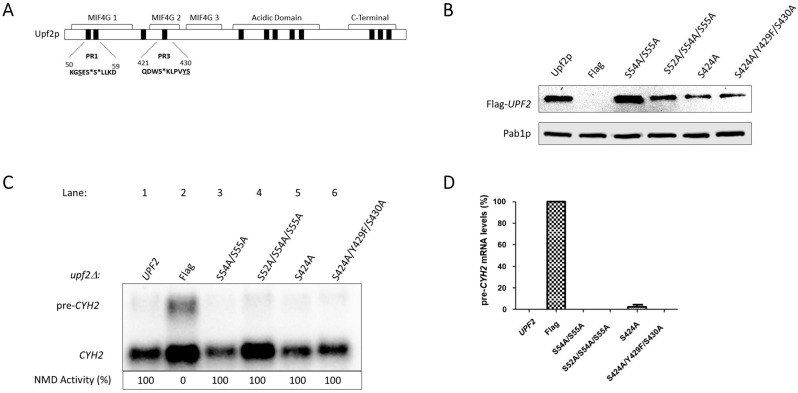
NMD activity does not require Upf2p mIF4G-1 and mIF4G-2 phosphorylated residues of either phospho-region 1 or phospho-region 3. (**A**) Illustrative representation of Upf2 phospho-region (PR) mutants 1 and 3. Phosphorylated residues S54, S55 and S424 are depicted with an asterisk (*) while adjacent potential phosphorylated residues S52, Y429, S430 are underlined. (**B**) Analysis of cytoplasmic extracts by Western blot showing Upf2 expression. Poly(A) binding protein (Pab1) was used as a loading control. (**C**) Northern blot from total cellular RNA was used to determine NMD activity of the Upf2 phosphorylated residues substitutions. (**D**) *pre-CYH2* mRNA accumulation from Northern blot expressed as a mean value ± standard deviation.

Examination of phospho-region 1 showed a serine, S52 (Figure 5A), adjacent to the serine we found to be phosphorylated in this study, S54 and S55 (Figure 3A). Likewise, examination of phospho-region 3 showed a tyrosine and serine, Y429 and S430 (Figure 5A), adjacent to the phosphorylated residue S424 identified here (Figure 3A). These adjacent residues were not identified as phosphorylated residues in our mass spectrometry analysis (Figure 2B). Nonetheless, these amino acid residues may potentially be phosphorylated under different conditions or phosphorylated at levels in which our analysis cannot detect. To test the role of the phosphorylated residues identified and the adjacent residues, single, double, and triple mutants were generated from the Flag-*UPF2* allele plasmid vector using site-directed mutagenesis (Figure 7A).

**Figure 8:**
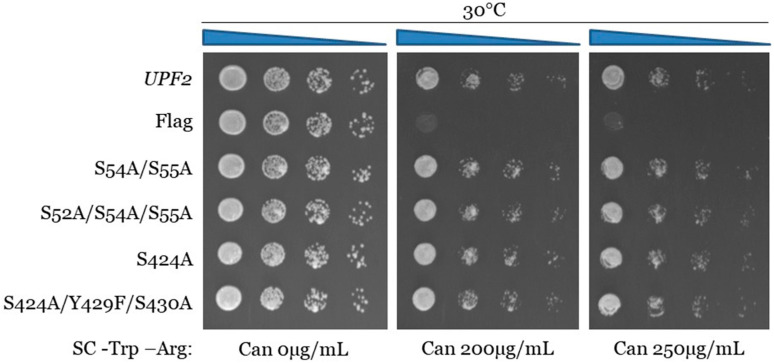
Proper translation termination does not require Upf2p mIF4G-1 and mIF4G-2 phosphorylated residues of either phospho-region 1 or phospho-region 3. *can1-100* nonsense suppression assay was used to assess the role of Upf2p in translation termination efficiency. Wild-type and mutant *upf2* yeast strains were serially diluted (1:10) five times. Each yeast strain was spotted on SC-Trp-Arg plates, each supplemented with varying concentrations of canavanine (0, 200, or 250 µg/ mL), followed by incubation at 30 °C for a duration of two days.

The serine and tyrosine residues were mutated to alanine and phenylalanine, respectively, to mimic unphosphorylated versions of these amino acids. One single point mutant (S424A), one double mutant (S54A/S55A), and two triple mutants (S52A/S54A/S55A and S424/Y429F/S430A) were generated. Expression of these *upf2* mutants was confirmed by Western blotting (Figure 7B). Northern blot analysis of total cellular RNA isolated from WT and the five mutant strains were used to determine NMD activity as described previously. As anticipated, NMD was not impaired, as its activity in the wild-type strain was 100%, while no activity was observed in the *upf2*Δ strain (Figure 7C, compare lanes 1 and 2). All the mutant constructs retain NMD activity at a level like the WT (Figure 7C,D; compare lane 1 with lanes 3, 4, 5 and 6). Likewise, none of the mutant strains showed a decrease in growth in the presence of canavanine (Figure 8 and Figure S3). Taken together, these data suggest that both NMD activity and translation termination accuracy are independent of the phosphorylation of S52, S54, and S424. In addition, nearby residues S52, Y429, and S430 do not have a redundant phosphorylation role in the absence of phosphorylated residues S54, S55, and S424, and are also not essential for NMD activity and translation termination.

### 3.4. Unphosphorylated Residues within Upf2p mIF4G-1 and mIF4G-2 Are Essential for NMD and Translation Termination Accuracy

**Figure 9:**
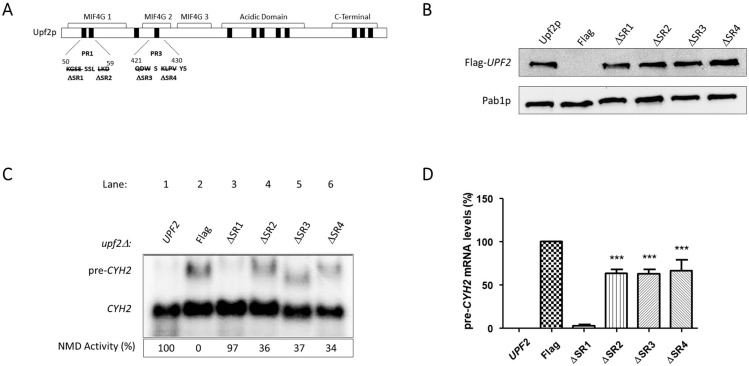
NMD activity requires Upf2p mIF4G-1 and mIF4G-2 non-phosphorylated segments. (**A**) Illustrative representation of Upf2p non-phosphorylated segments (SR) within phospho-region (PR) 1 and 3. Depicted by a strikethrough, in segment 1 (SR1) residues K50–E53 were deleted, in segment 2 (SR2) residues L57–D59 were deleted, segment 3 (SR3) residues Q42–W423, and in segment 4 (SR4) residues K425–V428 were removed. (**B**) Analysis of cytoplasmic extracts by Western blot showing Upf2 expression. Poly(A) binding protein (Pab1) was used as a loading control. (**C**) Northern blot from total cellular RNA was used to determine NMD activity of the Upf2p non-phosphorylated segments deletions. (**D**) *pre-CYH2* mRNA accumulation from Northern blot expressed as a mean value ± standard deviation. Significance of results, when compared to the WT strain, are represented by asterisks (*** *p* < 0.001).

**Figure 10:**
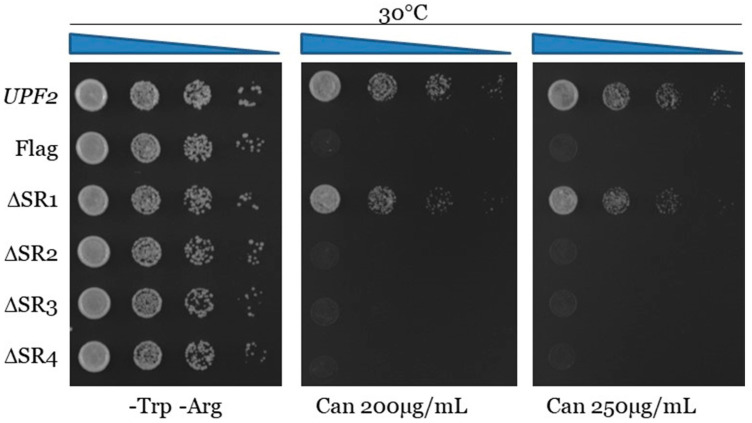
Proper translation termination requires Upf2p mIF4G-1 and mIF4G-2 non-phosphorylated segments. *can1-100* nonsense suppression assay was used to assess the role of Upf2p in translation termination efficiency. Wild-type and mutant *upf2* yeast strains were serially diluted (1:10) five times. Each yeast strain was spotted on SC-Trp-Arg plates, each supplemented with varying concentrations of canavanine (0, 200, or 250 µg/mL), followed by incubation at 30 °C for a duration of two days.

The activity of the studied regions appeared to be independent of their phosphorylation status, but, since the absence of these regions diminishes NMD activity, which might be due to impairments in the structure of Upf2p, we sought to further characterize them by studying other segments within them. To assess their role in NMD, two segments containing up to a maximum of four residues were deleted from each mIF4G region of the Flag-*UPF2* allele plasmid vector. The deleted segments were Segment 1) (K50–E53), Segment 2) (L57–D59) from phospho-region 1 in mIF4G-1, Segment 3) (Q421–W423), and Segment 4) (K425–V428) from phospho-region 3 in mIF4G-2. Constructs harboring these deletions were transformed into an *upf2*Δ strain (Figure 9A). Expression of these *upf2* mutants was confirmed by Western blotting (Figure 9B). The WT and mutant strains were subjected to Northern blot analysis to determine the NMD activity as described above. Levels of *pre-CYH2* mRNA showed a reduction of NMD activity of 64%, 63% and 66% for mutants lacking residues L57–D59, Q421–W423 and K425–V428, respectively (Figure 9C,D; lane 4–6). In contrast, deletion of residues K50–E53 had no significant effect on NMD activity (Figure 9C,D; lane 3). Results from the *can1-100* nonsense suppression assays show that deletion of segment 1 (K50–E53) did not present a nonsense suppression phenotype like the wild-type *UPF2* strain (Figure 10 and Figure S4). In contrast, strains harboring the deletion of segment 2 (L57–D59), segment 3 (Q421–W423), and segment 4 (K425–V428) exhibited reduced growth in the presence of canavanine, pointing to a decrease in translation termination efficiency (Figure 10 and Figure S4). All together, these data suggest that the regulation of NMD is modulated by non-phosphorylated residues residing within segment 2 (L57–D59) of mIF4G-1, and within segments 3 (Q421–W423) and 4 (K425–V428) of mIF4G-2.

### 3.5. Upf2p mIF4G-1 Residue D59 Is Required for NMD and Translation Termination Accuracy

**Figure 11:**
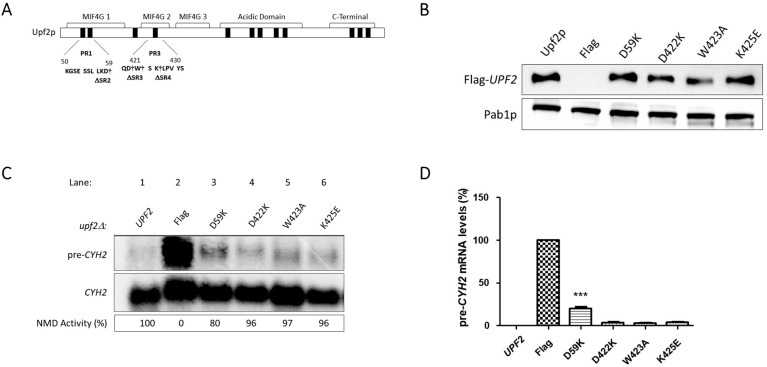
NMD activity requires Upf2p mIF4G-1 residue D59. (**A**) Illustrative representation of Upf2p sub-region mutants. Residues D59, D422, W423, and K425, which were substituted, are depicted with a dagger (†). (**B**) Analysis of cytoplasmic extracts by Western blot showing Upf2p expression. Poly(A) binding protein (Pab1) was used as a loading control. (**C**) Northern blot from total cellular RNA was used to determine NMD activity of the Upf2p single mutant deletions. (**D**) *pre-CYH2* mRNA accumulation from Northern blot expressed as a mean value ± standard deviation. Significance of results, when compared to the WT strain, are represented by asterisks (*** *p* < 0.001).

**Figure 12:**
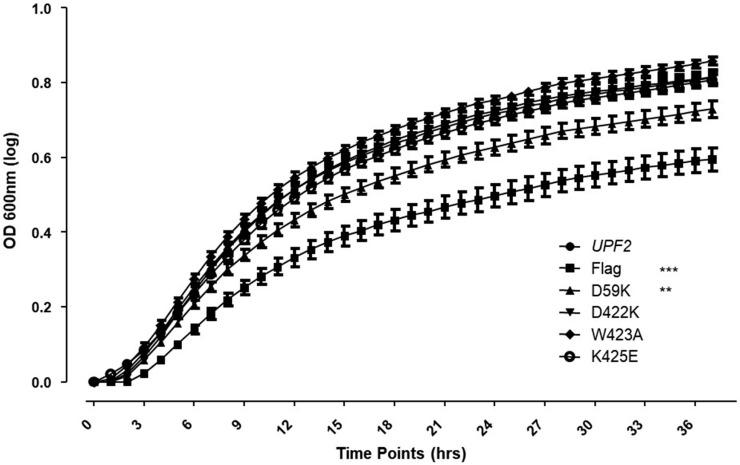
Proper translation termination requires Upf2p mIF4G-1 residue D59. *can1-100* nonsense suppression assay growth curves. Two-way ANOVA was used for statistical analysis. Significance of results, when compared to the WT strain, are represented by asterisks (** *p* < 0.01, *** *p* < 0.001).

Effects seen on NMD regulation when mIF4G segments 2, 3, and 4 were deleted may be due to an impairment of Upf2 structure. To discard this possibility, we tested the role of the essential residues within these segments of mIF4G-1 and mIF4G-2 through single mutants generated using site-directed mutagenesis on the Flag-*UPF2* allele plasmid vector (Figure 11A). Charged residues were mutated to incorporate charge inversions. Negatively charged residues D59 and D422 were substituted by lysine generating D59K and D422K. The positively charged residue K425 was substituted by glutamic acid generating K425E, while neutral residue W423 was substituted by alanine generating W423A. The expression of these mutants was confirmed by Western blotting (Figure 11B). Total cellular RNA isolated from WT and the five different point mutant strains were analyzed by Northern blot and NMD activity was ascertained as described above. Levels of *pre-CYH2* mRNA showed a 20% reduction of NMD activity for the point mutant D59K (Figure 11C,D; lane 3). In contrast, point mutants D422K, W423A and K425E had no significant effect on NMD activity. (Figure 11C,D; lane 4–6). Likewise, the point mutant D59K showed a decrease in growth when grown in the presence of canavanine (Figure 12 and Figure S5). Taken together, these data suggest that the regulation of NMD is influenced by the residue D59 residing within the mIF4G-1 of Upf2p.

## 4. Discussion

The mIF4G domains of Upf2p, which play a crucial role in regulating NMD activity, have been an overlooked area of research particularly in the context of protein–protein interactions. In *S. cerevisiae*, Upf3p has been shown to physically interact with the Upf2p mIF4G-3 in a way that is essential for NMD activity [22,55]. The Upf2p mIF4G-1 domain is also involved in mediating protein–protein interactions with Hrp1, a protein with canonical RNA recognition motifs (RRMs) [4,56,57] which is also important for maintaining normal levels of NMD activity [4]. Likewise, human Upf2 mIF4G-3 interacts with the Upf3 RNA recognition motif (RRM) via charged residues that abolish the Upf2–Upf3b interaction when mutated and leads to inhibition of NMD [21,22,58]. Upf2 mIF4G-3 has also been previously shown to interact, less strongly than Upf3, with eRF3, thus Upf2 functions in translation termination [59]. Examining the specific mIF4G domains of Upf2p and their potential variations across species is essential as it can offer a comprehensive understanding of the NMD regulatory network.

In this article we have identified twelve phosphorylated residues in *S. cerevisiae* Upf2p of which two are in the mIF4G-1 domain (S54 and S55) and one in the mIF4G-2 (S424) (Figure 3A,B). Our mutation analysis showed that phosphorylated residues within these domains do not disrupt NMD activity (Figure 7C,D). We have also identified two regions consisting of ten residues each (residues 50–59 and 421–430) harbored within Upf2p mIF4G-1 and Upf2 mIF4G-2, respectively, which are essential for function of the NMD mechanism (Figure 5C,D). By generating smaller deletions within these regions we demonstrated that the deletion of amino acids segments containing a minimum of three residues within mIF4G-1 and mIF4G-2 was sufficient to reduce the activity of NMD (Figure 9C,D).

Significantly, our charge inversion mutation analysis revealed an important role of the highly conserved negative charge residue D59 (Figure 4) in influencing NMD activity (Figure 11C,D). This residue is in helix 2 of the mIF4G-1, a region enriched in basic residues as shown in previous studies [42]. It engages with helix 1 of the mIF4G-1 through the residues R36 and K43, both of which have been identified as crucial for NMD activity [4,42]. Mutation of D59K significantly reduced NMD activity (Figure 11C,D), but not to the same extent as the *upf2*Δ strain. This reduction of NMD activity does not seem to be the result of an instability of Upf2p harboring the charge inversion mutant D59K since expression levels of it are approximately the same as the wild type (Figure 11B). Yet, our results are consistent with studies in which charge inversion of mIF4G-1 domain residues K35E/R36E and K42E/K43E/K45E in helix 1 caused an inhibition of NMD activity [42]. It remains unclear whether charge inversion mutation of D59K, or any of the residues previously identified as crucial for NMD activity through charge inversion mutation analysis [42], causes sufficient destabilization to alter the mIF4G-1 domain structure. Due to this, it remains of interest to further determine whether the observed effect in NMD activity is due to the loss of the D59 residue or to the destabilization of the mIF4G-1 by the introduction of a positive charge in this position. Hence, forthcoming experiments should prioritize examining the impact of a conventional D59A substitution, a double charge inversion mutant involving K43 and D59, and a triple charge inversion mutant encompassing R36, K43, and D59. This comprehensive approach aims to restore interactions that may be otherwise lost through individual inversions. Supported by other findings where changes in the electrical charges of amino acids resulted in a reduction of Upf2-binding affinity [60], we posit that the mIF4G domains may play a crucial role in shaping the specificity and affinity of interactions between Upf2p and as-yet-unidentified proteins associated with NMD, achieved through the evolutionary conservation of charged residues. Hence, a more in-depth examination of the mIF4G domains in Upf2p is imperative for elucidating their regulatory significance in NMD, whether it be by unveiling their influence through distinct protein–protein interactions or other yet-to-be-uncovered specific mechanisms.

Along with NMD in mammals and yeast, Upf2 promotes proper translation termination by repressing read-through of the premature termination codons [47,61]. Our results reveal a role for Upf2p mIF4G-1 and mIF4G-2 in translation termination accuracy, suggesting that this event also depends on these regions (Figure 5 and Figure 9). In addition, we showed that residue D59 located in the mIF4G-1, which plays a role in NMD, is also involved in the fidelity of translation termination (Figure 12). Future research should delve deeper into the interplay between these domains and other proteins involved in translation termination. Hence, we propose that disrupting the evolutionary conserved charged residues in the mIF4G-1 domains interfere with their ability to interaction with other proteins necessary for translation termination.

Finally, this study should be viewed as an initial step, emphasizing the need for additional research on both Upf2p phosphorylation and mIF4G domains within the broader cellular context. Also, even though the identification of specific residues within Upf2p mIF4G-1 is a significant contribution, further experiments such as X-ray crystallography or cryo-electron microscopy, which involve larger structural elements, are necessary to discern whether changes in NMD activity and translation termination accuracy are attributed solely to the identified residues or structural alterations. These structural analyses, especially focusing in the mIF4G domains evolutionary conserved charged residues, would enhance our comprehension of how these domains contribute to the regulatory mechanisms of NMD. As such, the intricate nature of the mIF4G domains and the potential for context-specific effects on normal mRNA transcripts and long non-coding RNAs remain unexplored and could have an impact on additional layers of regulatory functions.

## 5. Conclusions

**Figure 13:**
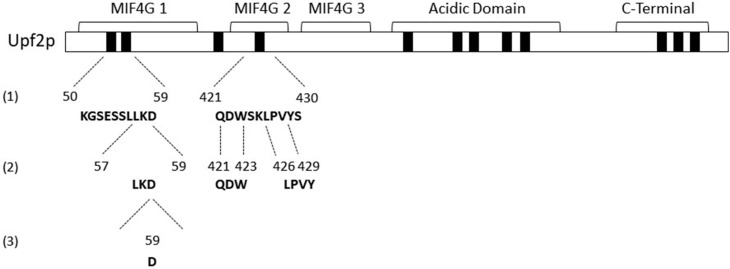
Characterization of *S. cerevisiae* Upf2p mIF4G domains. NMD activity requires Upf2p regions and segments within mIF4G-1 and mIF4G-2 and a residue within mIF4G-1. Schematic representation of Upf2p mIF4G domains characterization. mIF4G-1 and mIF4G-2 regions and segments required for NMD are aligned with the numbers (1) and (2), respectively. Single residue within mIF4G-1 required for NMD is aligned with the number (3).

Herein we have characterized Upf2p mIF4G-1 and mIF4G-2 domains providing evidence regarding their role in NMD and translation termination accuracy (Figure 13). Characterization of mIF4G-1 proved to be of particular importance since we were able to identify a region of ten residues, a segment of three residues (L57–D59), and a single residue (D59) capable of causing a significant reduction in the activity of NMD. While our findings shed light on the intricate role of Upf2p mIF4G-1 and mIF4G-2 domains in coordinating NMD and translation termination accuracy, further research is needed to explore the broader landscape of Upf2p mIF4G domains and understand its regulatory network.

## Data Availability

The data presented in this study are available in the body of the article, supplementary materials and at figshare (Colon, Edgardo (2023). Original Images of Blots and Gels For Article “Characterization of the mIF4G domains in the RNA surveillance protein Upf2p”. figshare. Collection. https://doi.org/10.6084/m9.figshare.c.6930301.v1 Accessed on 14 November 2023).

## Supplementary Materials

The following supporting information can be downloaded at: https://www.mdpi.com/article/10.3390/cimb46010017/s1, Figure S1. Example of MS/MS spectrum for precursor ion GEDEDAEISpTPNTESAPGK; Figure S2. Proper translation termination requires Upf2 mIF4G-1 and mIF4G-2 phosphorylated regions 1 and 3; Figure S3. Proper translation termination does not require Upf2 mIF4G-1 and mIF4G-2 phosphorylated residues of either phospho-region 1 or phospho-region 3; Figure S4. Proper translation termination requires Upf2 mIF4G-1 and mIF4G-2 non-phosphorylated segments; Figure S5. Proper translation termination requires Upf2 mIF4G-1 residue D59.

## References

[1] Gonzalez C.I., Wang W., Peltz S.W. (2001). Nonsense-mediated mRNA decay in Saccharomyces cerevisiae: A quality control mechanism that degrades transcripts harboring premature termination codons. Cold Spring Harb. Symp. Quant. Biol..

[2] Amrani N., Dong S., He F., Ganesan R., Ghosh S., Kervestin S., Li C., Mangus D.A., Spatrick P., Jacobson A. (2006). Aberrant termination triggers nonsense-mediated mRNA decay. Biochem. Soc. Trans..

[3] Behm-Ansmant I., Kashima I., Rehwinkel J., Sauliere J., Wittkopp N., Izaurralde E. (2007). mRNA quality control: An ancient machinery recognizes and degrades mRNAs with nonsense codons. FEBS Lett..

[4] Wang W., Cajigas I.J., Peltz S.W., Wilkinson M.F., Gonzalez C.I. (2006). Role for Upf2p phosphorylation in Saccharomyces cerevisiae nonsense-mediated mRNA decay. Mol. Cell Biol..

[5] Kervestin S., Jacobson A. (2012). NMD: A multifaceted response to premature translational termination. Nat. Rev. Mol. Cell Biol..

[6] Schweingruber C., Rufener S.C., Zund D., Yamashita A., Muhlemann O. (2013). Nonsense-mediated mRNA decay—Mechanisms of substrate mRNA recognition and degradation in mammalian cells. Biochim. Biophys. Acta.

[7] Monaghan L., Longman D., Caceres J.F. (2023). Translation-coupled mRNA quality control mechanisms. EMBO J..

[8] Andjus S., Morillon A., Wery M. (2021). From Yeast to Mammals, the Nonsense-Mediated mRNA Decay as a Master Regulator of Long Non-Coding RNAs Functional Trajectory. Noncoding RNA.

[9] Palacios I.M. (2013). Nonsense-mediated mRNA decay: From mechanistic insights to impacts on human health. Brief. Funct. Genom..

[10] Rebbapragada I., Lykke-Andersen J. (2009). Execution of nonsense-mediated mRNA decay: What defines a substrate?. Curr. Opin. Cell Biol..

[11] Wittmann J., Hol E.M., Jack H.M. (2006). hUPF2 silencing identifies physiologic substrates of mammalian nonsense-mediated mRNA decay. Mol. Cell Biol..

[12] Guan Q., Zheng W., Tang S., Liu X., Zinkel R.A., Tsui K.W., Yandell B.S., Culbertson M.R. (2006). Impact of nonsense-mediated mRNA decay on the global expression profile of budding yeast. PLoS Genet..

[13] Alonso C.R. (2005). Nonsense-mediated RNA decay: A molecular system micromanaging individual gene activities and suppressing genomic noise. BioEssays News Rev. Mol. Cell. Dev. Biol..

[14] Hug N., Longman D., Caceres J.F. (2016). Mechanism and regulation of the nonsense-mediated decay pathway. Nucleic Acids Res..

[15] Celik A., Baker R., He F., Jacobson A. (2017). High-resolution profiling of NMD targets in yeast reveals translational fidelity as a basis for substrate selection. RNA.

[16] Malabat C., Feuerbach F., Ma L., Saveanu C., Jacquier A. (2015). Quality control of transcription start site selection by nonsense-mediated-mRNA decay. eLife.

[17] Garcia-Martinez J., Singh A., Medina D., Chavez S., Perez-Ortin J.E. (2023). Enhanced gene regulation by cooperation between mRNA decay and gene transcription. Biochim. Biophys. Acta Gene Regul. Mech..

[18] Chang Y.F., Imam J.S., Wilkinson M.F. (2007). The nonsense-mediated decay RNA surveillance pathway. Annu. Rev. Biochem..

[19] Lykke-Andersen J., Shu M.D., Steitz J.A. (2000). Human Upf proteins target an mRNA for nonsense-mediated decay when bound downstream of a termination codon. Cell.

[20] Mendell J.T., Medghalchi S.M., Lake R.G., Noensie E.N., Dietz H.C. (2000). Novel Upf2p orthologues suggest a functional link between translation initiation and nonsense surveillance complexes. Mol. Cell Biol..

[21] Kadlec J., Izaurralde E., Cusack S. (2004). The structural basis for the interaction between nonsense-mediated mRNA decay factors UPF2 and UPF3. Nat. Struct. Mol. Biol..

[22] He F., Brown A.H., Jacobson A. (1997). Upf1p, Nmd2p, and Upf3p are interacting components of the yeast nonsense-mediated mRNA decay pathway. Mol. Cell Biol..

[23] Chamieh H., Ballut L., Bonneau F., Le Hir H. (2008). NMD factors UPF2 and UPF3 bridge UPF1 to the exon junction complex and stimulate its RNA helicase activity. Nat. Struct. Mol. Biol..

[24] He F., Jacobson A. (1995). Identification of a novel component of the nonsense-mediated mRNA decay pathway by use of an interacting protein screen. Genes Dev..

[25] Ruiz-Echevarria M.J., Gonzalez C.I., Peltz S.W. (1998). Identifying the right stop: Determining how the surveillance complex recognizes and degrades an aberrant mRNA. EMBO J..

[26] Carter M.S., Li S., Wilkinson M.F. (1996). A splicing-dependent regulatory mechanism that detects translation signals. EMBO J..

[27] Kervestin S., Li C., Buckingham R., Jacobson A. (2012). Testing the faux-UTR model for NMD: Analysis of Upf1p and Pab1p competition for binding to eRF3/Sup35p. Biochimie.

[28] Amrani N., Ganesan R., Kervestin S., Mangus D.A., Ghosh S., Jacobson A. (2004). A faux 3’-UTR promotes aberrant termination and triggers nonsense-mediated mRNA decay. Nature.

[29] Lejeune F., Maquat L.E. (2005). Mechanistic links between nonsense-mediated mRNA decay and pre-mRNA splicing in mammalian cells. Curr. Opin. Cell Biol..

[30] Gehring N.H., Kunz J.B., Neu-Yilik G., Breit S., Viegas M.H., Hentze M.W., Kulozik A.E. (2005). Exon-junction complex components specify distinct routes of nonsense-mediated mRNA decay with differential cofactor requirements. Mol. Cell.

[31] Hwang H.J., Park Y., Kim Y.K. (2021). UPF1: From mRNA Surveillance to Protein Quality Control. Biomedicines.

[32] Lejeune F. (2022). Nonsense-Mediated mRNA Decay, a Finely Regulated Mechanism. Biomedicines.

[33] Clarke L.A., Luz V.C.C., Targowski S., Ramalho S.S., Farinha C.M., Amaral M.D. (2021). Integrity and Stability of PTC Bearing CFTR mRNA and Relevance to Future Modulator Therapies in Cystic Fibrosis. Genes.

[34] Vallverdu-Prats M., Brugada R., Alcalde M. (2022). Premature Termination Codon in 5’ Region of Desmoplakin and Plakoglobin Genes May Escape Nonsense-Mediated Decay through the Reinitiation of Translation. Int. J. Mol. Sci..

[35] Chakrabarti S., Jayachandran U., Bonneau F., Fiorini F., Basquin C., Domcke S., Le Hir H., Conti E. (2011). Molecular mechanisms for the RNA-dependent ATPase activity of Upf1 and its regulation by Upf2. Mol. Cell.

[36] Kim Y.K., Maquat L.E. (2019). UPFront and center in RNA decay: UPF1 in nonsense-mediated mRNA decay and beyond. RNA.

[37] Xue G., Maciej V.D., Machado de Amorim A., Pak M., Jayachandran U., Chakrabarti S. (2023). Modulation of RNA-binding properties of the RNA helicase UPF1 by its activator UPF2. RNA.

[38] Clerici M., Mourao A., Gutsche I., Gehring N.H., Hentze M.W., Kulozik A., Kadlec J., Sattler M., Cusack S. (2009). Unusual bipartite mode of interaction between the nonsense-mediated decay factors, UPF1 and UPF2. EMBO J..

[39] Ponting C.P. (2000). Novel eIF4G domain homologues linking mRNA translation with nonsense-mediated mRNA decay. Trends Biochem. Sci..

[40] Clerici M., Deniaud A., Boehm V., Gehring N.H., Schaffitzel C., Cusack S. (2014). Structural and functional analysis of the three MIF4G domains of nonsense-mediated decay factor UPF2. Nucleic Acids Res..

[41] Aravind L., Koonin E.V. (2000). Eukaryote-specific domains in translation initiation factors: Implications for translation regulation and evolution of the translation system. Genome Res..

[42] Fourati Z., Roy B., Millan C., Coureux P.D., Kervestin S., van Tilbeurgh H., He F., Uson I., Jacobson A., Graille M. (2014). A highly conserved region essential for NMD in the Upf2 N-terminal domain. J. Mol. Biol..

[43] Wang J., Wilkinson M.F. (2001). Deletion mutagenesis of large (12-kb) plasmids by a one-step PCR protocol. BioTechniques.

[44] Makarova O., Kamberov E., Margolis B. (2000). Generation of deletion and point mutations with one primer in a single cloning step. BioTechniques.

[45] Gietz D., St Jean A., Woods R.A., Schiestl R.H. (1992). Improved method for high efficiency transformation of intact yeast cells. Nucleic Acids Res..

[46] Herrick D., Parker R., Jacobson A. (1990). Identification and comparison of stable and unstable mRNAs in Saccharomyces cerevisiae. Mol. Cell Biol..

[47] Maderazo A.B., He F., Mangus D.A., Jacobson A. (2000). Upf1p control of nonsense mRNA translation is regulated by Nmd2p and Upf3p. Mol. Cell Biol..

[48] Cui Y., Hagan K.W., Zhang S., Peltz S.W. (1995). Identification and characterization of genes that are required for the accelerated degradation of mRNAs containing a premature translational termination codon. Genes Dev..

[49] Kaufer N.F., Fried H.M., Schwindinger W.F., Jasin M., Warner J.R. (1983). Cycloheximide resistance in yeast: The gene and its protein. Nucleic Acids Res..

[50] Ahmad M., Bussey H. (1986). Yeast arginine permease: Nucleotide sequence of the CAN1 gene. Curr. Genet..

[51] He F., Peltz S.W., Donahue J.L., Rosbash M., Jacobson A. (1993). Stabilization and ribosome association of unspliced pre-mRNAs in a yeast upf1- mutant. Proc. Natl. Acad. Sci. USA.

[52] Lasalde C., Rivera A.V., Leon A.J., Gonzalez-Feliciano J.A., Estrella L.A., Rodriguez-Cruz E.N., Correa M.E., Cajigas I.J., Bracho D.P., Vega I.E. (2014). Identification and functional analysis of novel phosphorylation sites in the RNA surveillance protein Upf1. Nucleic Acids Res..

[53] Estrella L.A., Wilkinson M.F., Gonzalez C.I. (2009). The shuttling protein Npl3 promotes translation termination accuracy in Saccharomyces cerevisiae. J. Mol. Biol..

[54] Ono B.I., Ishino Y., Shinoda S. (1983). Nonsense mutations in the can1 locus of Saccharomyces cerevisiae. J. Bacteriol..

[55] Serin G., Gersappe A., Black J.D., Aronoff R., Maquat L.E. (2001). Identification and characterization of human orthologues to Saccharomyces cerevisiae Upf2 protein and Upf3 protein (Caenorhabditis elegans SMG-4). Mol. Cell Biol..

[56] Gonzalez C.I., Ruiz-Echevarria M.J., Vasudevan S., Henry M.F., Peltz S.W. (2000). The yeast hnRNP-like protein Hrp1/Nab4 marks a transcript for nonsense-mediated mRNA decay. Mol. Cell.

[57] Henry M., Borland C.Z., Bossie M., Silver P.A. (1996). Potential RNA binding proteins in Saccharomyces cerevisiae identified as suppressors of temperature-sensitive mutations in NPL3. Genetics.

[58] Kashima I., Yamashita A., Izumi N., Kataoka N., Morishita R., Hoshino S., Ohno M., Dreyfuss G., Ohno S. (2006). Binding of a novel SMG-1-Upf1-eRF1-eRF3 complex (SURF) to the exon junction complex triggers Upf1 phosphorylation and nonsense-mediated mRNA decay. Genes Dev..

[59] Lopez-Perrote A., Castano R., Melero R., Zamarro T., Kurosawa H., Ohnishi T., Uchiyama A., Aoyagi K., Buchwald G., Kataoka N. (2016). Human nonsense-mediated mRNA decay factor UPF2 interacts directly with eRF3 and the SURF complex. Nucleic Acids Res..

[60] Bufton J.C., Powers K.T., Szeto J.A., Toelzer C., Berger I., Schaffitzel C. (2022). Structures of nonsense-mediated mRNA decay factors UPF3B and UPF3A in complex with UPF2 reveal molecular basis for competitive binding and for neurodevelopmental disorder-causing mutation. Nucleic Acids Res..

[61] Wang W., Czaplinski K., Rao Y., Peltz S.W. (2001). The role of Upf proteins in modulating the translation read-through of nonsense-containing transcripts. EMBO J..

