# Supplementary material for: Characterization of the mIF4G Domains in the RNA Surveillance Protein Upf2p
Source: Curr Issues Mol Biol. 2023 Dec 29;46(1):244–61. doi: 10.3390/cimb46010017 (PMC10814901; doi:10.3390/cimb46010017)
Supplement: Supplementary file 1 [file cimb-46-00017-s001.zip › cimb-2749036-supplementary.pdf]

current issues in
molecular biology

MDPI

Supplementary Data

Characterization of the mIF4G domains in the RNA surveillance protein Upf2p

Edgardo M. Colón[1,2*], Luis A. Haddock III[1,2], Clarivel Lasalde[1], Qishan Lin[3,4], Juan S. Ramírez-Lugo[1] and Carlos I. González[1,2*]

Supplementary Figures

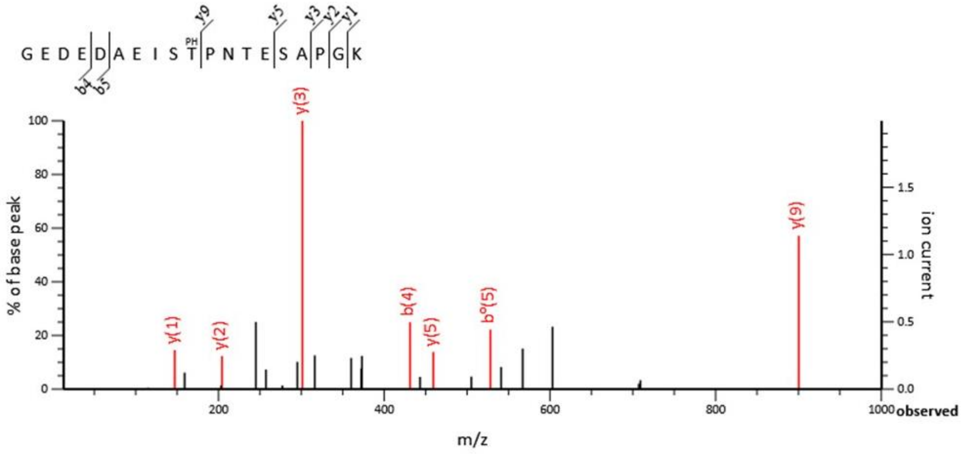

Figure S1. Example of MS/MS spectrum for precursor ion GEDEDAEISpTPNTESAPGK. This precursor ion was selected and subjected to fragmentation, generating b and y product ions that represent specific fragments used for identification of the peptide sequence and phosphorylation sites. Loss of H_3PO_4 from *y9* fragment after β-elimination was evidently observed.

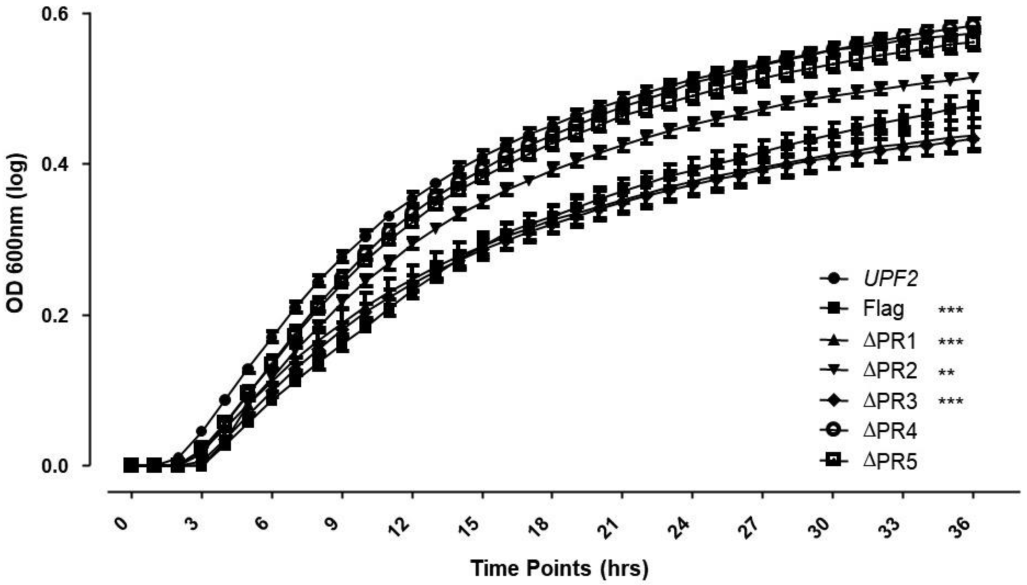

Figure S2. Proper translation termination requires Upf2p mIF4G-1 and mIF4G-2 phosphorylated regions 1 and 3. *can1-100* nonsense suppression assay growth curves. Two-way ANOVA was used for statistical analysis. Significance of results, when compared to the WT strain, are represented by asterisks (**$p < 0.01$, ***$p < 0.001$).

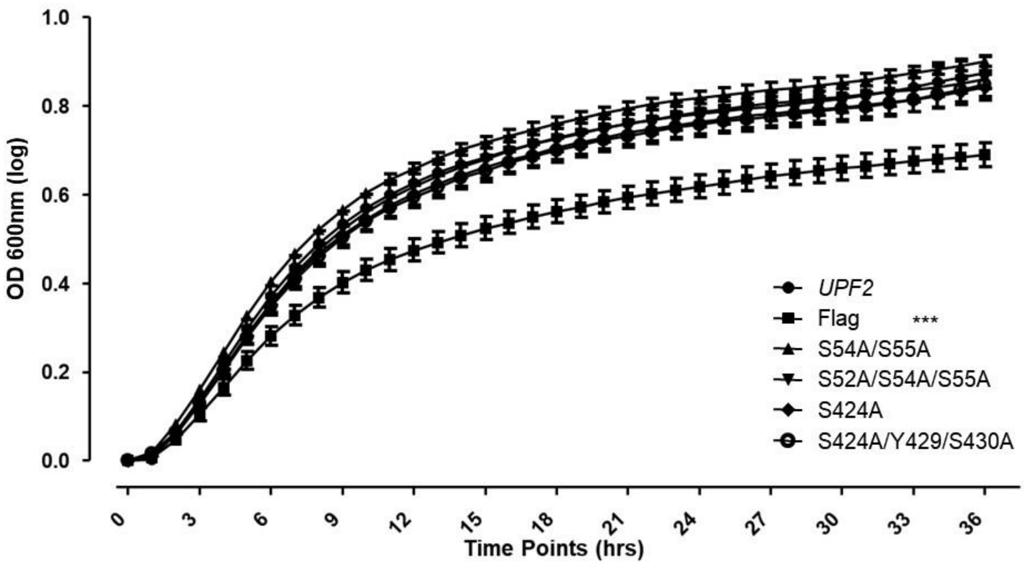

Figure S3. Proper translation termination does not require Upf2p mIF4G-1 and mIF4G-2 phosphorylated residues of either phospho-region 1 or phospho-region 3. *can1-100* nonsense suppression assay growth curves. Two-way ANOVA was used for statistical analysis. Significance of results, when compared to the WT strain, are represented by asterisks (***p < 0.001).

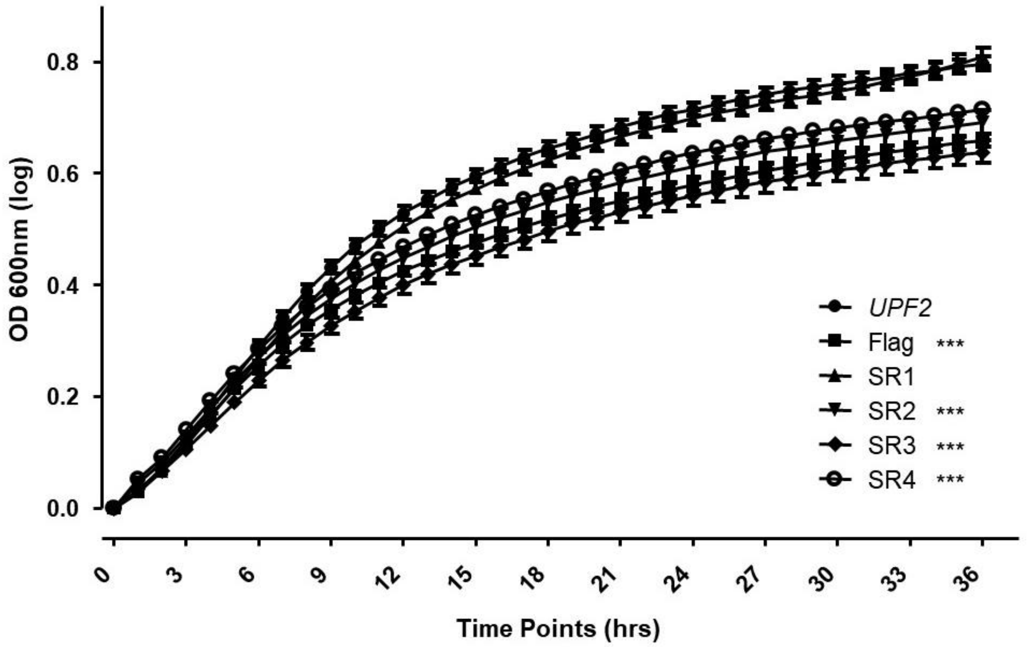

Figure S4. Proper translation termination requires Upf2p mIF4G-1 and mIF4G-2 non-phosphorylated segments. *can1-100* nonsense suppression assay growth curves. Two-way ANOVA was used for statistical analysis. Significance of results, when compared to the WT strain, are represented by asterisks (***p < 0.001).

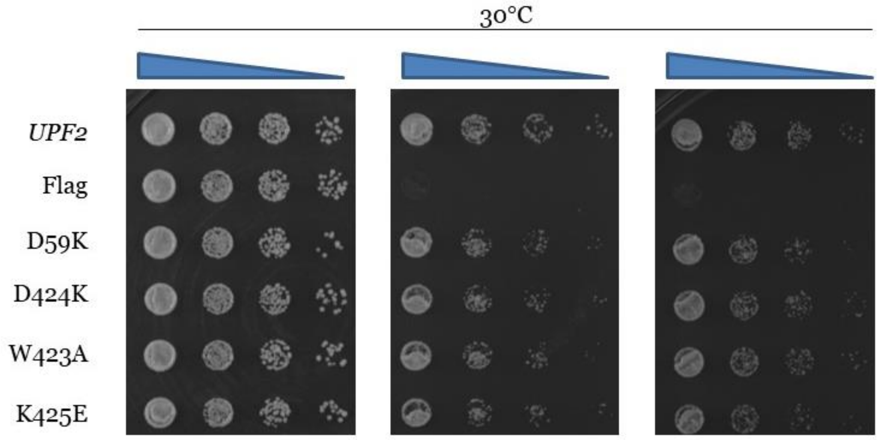

Figure S5. Proper translation termination requires Upf2p mIF4G-1 residue D59. *can1-100* nonsense suppression assay was used to assess the role of Upf2p in translation termination efficiency. Wild-type and mutant *upf2* yeast strains were serially diluted (1:10) five times.
